# Discrimination of the Veterans Aging Cohort Study Index 2.0 for Predicting Cause-specific Mortality Among Persons With HIV in Europe and North America

**DOI:** 10.1093/ofid/ofae333

**Published:** 2024-06-17

**Authors:** Julie Ambia, Suzanne M Ingle, Kathleen McGinnis, Nikos Pantazis, Michael J Silverberg, Linda Wittkop, Katharina Kusejko, Heidi Crane, Ard van Sighem, Mario Sarcletti, Alessandro Cozzi-Lepri, Pere Domingo, Inma Jarrin, Christoph Wyen, Mojgan Hessamfar, Lei Zhang, Matthias Cavassini, Juan Berenguer, Timothy R Sterling, Peter Reiss, Sophie Abgrall, M John Gill, Amy Justice, Jonathan A C Sterne, Adam Trickey

**Affiliations:** Population Health Sciences, University of Bristol, Bristol, UK; Population Health Sciences, University of Bristol, Bristol, UK; VA Connecticut Healthcare System, US Department of Veteran Affairs, West Haven, Connecticut, USA; Department of Hygiene, Epidemiology and Medical Statistics, Medical School, National and Kapodistrian University of Athens, Athens, Greece; Division of Research, Kaiser Permanente Northern California, Oakland, California, USA; University of Bordeaux, INSERM, Institut Bergonié, BPH, U1219, CIC-EC 1401, F-33000, Bordeaux, France; INRIA SISTM team, Talence. CHU de Bordeaux, Service d’information médicale, INSERM, Institut Bergonié, CIC-EC 1401, F-33000, Bordeaux, France; Division for Infectious Diseases and Hospital Epidemiology, University Hospital Zurich, University of Zurich, Zurich, Switzerland; Division of Infectious Diseases, Department of Medicine, University of Washington, Seattle, Washington, USA; Stichting HIV Monitoring, Amsterdam, Netherlands; Department of Dermatology and Venereology, Medical University of Innsbruck, Innsbruck, Austria; Institute for Global Health, University College London, London, UK; Department of Infectious Diseases, Santa Creu i Sant Pau Hospital, Barcelona, Spain; National Centre of Epidemiology, Carlos III Health Institute, Madrid, Spain; CIBER de Enfermedades Infecciosas, Instituto de Salud Carlos III, Madrid, Spain; Department I for Internal Medicine, University Hospital of Cologne, Cologne, Germany; Department of Internal Medicine and Infectious Disease, Bordeaux University Hospital F-33000, Bordeaux, France; Population Health Sciences, University of Bristol, Bristol, UK; School of Public Finance and Management, Yunnan University of Finance and Economics, Kunming, China; Infectious Diseases Service, Lausanne University Hospital and University of Lausanne, Lausanne, Switzerland; Hospital General Universitario Gregorio Marañón, IiSGM, CIBERINFEC, Madrid, Spain; Division of Infectious Diseases, Department of Medicine, Vanderbilt University School of Medicine, Nashville, Tennessee, USA; Stichting HIV Monitoring, Amsterdam, The Netherlands; Amsterdam UMC, University of Amsterdam, Global Health, Amsterdam, The Netherlands; Amsterdam Institute for Global Health and Development, Amsterdam, The Netherlands; APHP, Hôpital Béclère, Service de Médecine Interne, Clamart, France; APHP, Université Paris-Saclay, Univ. Paris-Sud, UVSQ, CESP INSERM U1018, Le Kremlin-Bicêtre, France; Department of Medicine, University of Calgary, South Alberta HIV Clinic, Calgary, Alberta, Canada; VA Connecticut Healthcare System, US Department of Veteran Affairs, West Haven, Connecticut, USA; Department of Internal Medicine, Yale University School of Medicine, New Haven, Connecticut, USA; Population Health Sciences, University of Bristol, Bristol, UK; Population Health Sciences, University of Bristol, Bristol, UK

**Keywords:** VACS, C-statistic, prognosis, absolute risk, multiple imputation

## Abstract

**Background:**

Predicting cause-specific mortality among people with HIV (PWH) could facilitate targeted care to improve survival. We assessed discrimination of the Veterans Aging Cohort Study (VACS) Index 2.0 in predicting cause-specific mortality among PWH on antiretroviral therapy (ART).

**Methods:**

Using Antiretroviral Therapy Cohort Collaboration data for PWH who initiated ART between 2000 and 2018, VACS Index 2.0 scores (higher scores indicate worse prognosis) were calculated around a randomly selected visit date at least 1 year after ART initiation. Missingness in VACS Index 2.0 variables was addressed through multiple imputation. Cox models estimated associations between VACS Index 2.0 and causes of death, with discrimination evaluated using Harrell's C-statistic. Absolute mortality risk was modelled using flexible parametric survival models.

**Results:**

Of 59 741 PWH (mean age: 43 years; 80% male), the mean VACS Index 2.0 at baseline was 41 (range: 0–129). For 2425 deaths over 168 162 person-years follow-up (median: 2.6 years/person), AIDS (n = 455) and non–AIDS-defining cancers (n = 452) were the most common causes. Predicted 5-year mortality for PWH with a mean VACS Index 2.0 score of 38 at baseline was 1% and approximately doubled for every 10-unit increase. The 5-year all-cause mortality C-statistic was .83. Discrimination with the VACS Index 2.0 was highest for deaths resulting from AIDS (0.91), liver-related (0.91), respiratory-related (0.89), non-AIDS infections (0.87), and non–AIDS-defining cancers (0.83), and lowest for suicides/accidental deaths (0.65).

**Conclusions:**

For deaths among PWH, discrimination with the VACS Index 2.0 was highest for deaths with measurable physiological causes and was lowest for suicide/accidental deaths.

## Teaser

For people with HIV on antiretroviral therapy, the Veterans Aging Cohort Study Index 2.0 better predicted AIDS-, liver-, and respiratory-related deaths than suicides/accidental deaths. Predicted 5-year mortality risks for each cause were low, except for people with very high Veterans Aging Cohort Study Index 2.0 scores.

## BACKGROUND

Mortality among people with HIV (PWH) has markedly decreased since the introduction of highly active antiretroviral therapy in the mid-1990s [1], driven by substantial declines in AIDS-related mortality [2]. However, mortality rates among PWH on antiretroviral therapy (ART) are still higher than in the general population [3]. Factors contributing to this include higher prevalence of comorbidities and behavioral risk factors such as smoking and substance use [4, 5].

The Veterans Aging Cohort Study (VACS) Index [6] was developed to discriminate all-cause mortality risk in PWH [7], accounting for interactions among organ dysfunctions, such as those in the liver and lungs, HIV, and comorbidities. The VACS Index 2.0 improved on discrimination of the original score through the addition of non–HIV-specific biomarkers (albumin, white blood cells, and body mass index) [8].

The VACS Index 2.0 has been applied to predict the risk of hospitalization [9], all-cause mortality [10], and functional decline [11] among PWH. The VACS Index 2.0 also acts as an indicator of physiologic frailty [12], neurocognitive impairment [13], and can predict the risk of hospitalization, intensive care unit admission, and mortality among PWH that had COVID-19 [14]. However, its utility in predicting cause-specific mortality among PWH has not been evaluated. This could enable the design of targeted interventions and tailored care plans to address risks associated with cause-specific mortality. We assessed the discrimination of VACS Index 2.0 in predicting specific causes of death among PWH and estimated 5-year risks of specific causes of death.

## METHODS

### Study Setting and Population

Data were from the Antiretroviral Therapy Cohort Collaboration (ART-CC) 2019 dataset, which includes 20 cohorts of adult PWH on ART in Europe and North America [15]. Cohorts were included if a cause of death was assigned to more than 70% of PWH who had died. PWH were included if they started combination ART (consisting of at least 3 ART drugs) from 2000 to 2018, were aged ≥16 years, and had survived for at least 1 year after starting ART.

### Coding Causes of Death

A single cause of death for each person who died was assigned using an adapted version of the Coding of Death in HIV protocol (CoDe) protocol [16]. Further details are described elsewhere [17]. In summary, a clinician used cause of death data (either in International Classification of Diseases [ICD]-9 or ICD-10 format, or as free-text) combined with demographic, medical, and laboratory data, to assign a cause of death using the CoDe categorizations. For deaths in ICD-9 or ICD-10 format, a computer algorithm also assigned a cause of death. For deaths in free-text format, a second clinician assigned a cause of death instead of the algorithm. Disagreements between the assigned causes of death were reviewed by a panel of clinicians until consensus was reached.

Deaths were grouped into 10 categories (Supplementary Table 1): AIDS, cardiovascular, respiratory, substance abuse, liver-related, non-AIDS infection, non–AIDS-defining cancers (NADCs), suicide/accidents, unclassifiable, and other deaths. Suicide/accidental deaths were grouped together, due to the common misclassification of suicides as accidents [18]. Hepatocellular carcinoma mortality was included in the liver-related, rather than NADCs category to include deaths related to viral hepatitis as liver related.

### Follow-up Time

For consistency with other research, the value of VACS Index 2.0 on a randomly selected visit date occurring at least 1 year after initiation of ART was used, with follow-up starting on that date [10]. This will be our baseline date for the analysis. The first year after ART initiation was excluded because many deaths during this period are due to PWH presenting to care with advanced disease [19]. Follow-up ended at the earliest of death, loss to follow-up, 5 years after baseline, or the cohort-specific administrative censoring date, the latest of which was June 2020.

### VACS Index 2.0

The VACS Index 2.0 is a weighted sum of 10 variables: age, traditional HIV indicators (CD4 cell count, HIV RNA), organ system injury indicators (hemoglobin, Fibrosis-4 Index, estimated glomerular filtration rate, albumin, white blood cell count), body mass index, and hepatitis C virus infection. We calculated validated composite biomarkers of liver and renal injury: Fibrosis-4 Index was calculated using aspartate aminotransferase, alanine aminotransferase, platelets, and age; estimated glomerular filtration rate was calculated via the CKD-EPI equation using serum creatinine, sex, and age [20]. Higher values of VACS Index 2.0 are indicative of poorer prognosis for mortality [10]. Laboratory test values were included in the analysis if measured within 365 days before or 7 days after the baseline date.

### Statistical Analyses

We derived Kaplan-Meier estimates of 5-year all-cause mortality. Hazard ratios (HR) for all-cause and cause-specific mortality per 10-unit increase in VACS Index 2.0 were estimated using Cox models. When the outcome of interest is cause-specific mortality the other causes of death represent a competing risk, which precludes the outcome of interest and can bias results. Therefore, we compared the Cox model HRs with subdistribution HRs estimated from competing risks regression models. Discrimination of VACS Index 2.0 in predicting cause-specific mortality was assessed using Harrell's C-statistic, which measures the ability of a predictive model to distinguish between individuals who do and do not experience the outcome and varies from 0.5 (no discrimination) to 1.0 (perfect discrimination) [21]. Analyses were additionally stratified into 2 calendar periods of ART initiation: 2000–2009 and 2010–2018. Subgroup analyses were conducted to assess the discrimination of the VACS Index 2.0 between men and women and between people of White and non-White ethnicity (combining non-White ethnicity into 1 category because of a lack of deaths). This division allowed for the examination of any potential differences in the predictive ability of the VACS Index 2.0 over time. However, subdividing the calendar periods further was not possible because of the limited number of deaths occurring after 2010.

To make mortality predictions more relevant to current mortality among PWH on ART, models to predict 5-year cause-specific mortality were restricted to PWH who initiated ART between 2010 and 2018 [22]. We used the Royston-Parmar flexible parametric survival model, which uses restricted cubic splines to flexibly model the baseline log-cumulative hazard [23]. The Bayesian Information Criterion was used to determine the optimal number of interior knots (1–4 knots were assessed) for the baseline spline function using an initial model fitted without any variables. Bayesian Information Criterion [24] was also used to determine the best functional form of the VACS Index 2.0 (Supplementary Figure 1, Supplementary Table 2, and 3).

### Missing Data

Univariable and multivariable logistic regression models were used to assess predictors of missingness. Multiple imputation by chained equations was performed with a fully conditional specification [25], incorporating all variables in the analysis model: VACS Index 2.0 components at baseline, calendar year of ART initiation, cohort, VACS Index 2.0 components at ART initiation, cause of death, and the Nelson-Aalen cumulative baseline hazard estimate [26]. Only VACS Index 2.0 components with missing values were imputed using multiple imputation by chained equations. One hundred imputed datasets were created, and Cox regression models were estimated from each. Effect estimates and discrimination statistics were pooled using Rubin's rules [27]. Further information on the multiple imputation process is included in the Appendix. A sensitivity analysis was performed to compare the C-statistic generated from complete cases to that generated from the imputed data. This work was carried out using the computational facilities of the Advanced Computing Research Centre, University of Bristol (http://www.bristol.ac.uk/acrc/). Analyses were conducted using Stata version 17.0 [28].

## RESULTS

Twelve ART-CC cohorts were eligible for inclusion based on completeness of cause of death data. 59 741 PWH from these cohorts contributed to analyses with 168 162 person-years of follow-up. The median follow-up time for each person was 2.6 (interquartile range [IQR]: 1.2–5.0) years. The mean age at baseline was 43 years, most (47 842; [80%]) were male, and more than half (31 287 [52.4%]) of those included were men who had acquired HIV through sex with men (Table 1). A total of 52 294 (90.2%) had viral load <100 copies/mL at baseline, whereas 10 679 (17.9%) had AIDS at ART initiation. VACS Index 2.0 scores at baseline ranged from 0 to 129.

**Table 1.** Characteristics at Follow-up Start of Included Persons With HIV

	2000–2018 n = 59 741 (100.0%)	2000–2009 n = 26 493 (44.3%)	2010–2018 n = 33 248 (55.7%)
Categorical characteristics: n (%)			
Male	47 842 (80.1%)	19 780 (74.7%)	28 062 (84.4%)
Missing	0	0	0
Hepatitis C virus (RNA positive)	5193 (8.7%)	3273 (12.4%)	1920 (5.8%)
Missing	0	0	0
AIDS at ART initiation	10 679 (17.9%)	6428 (24.3%)	4251 (12.8%)
Missing	0	0	0
Probable route of HIV acquisition			
Men who have sex with men	31 287 (52.4%)	11 044 (41.7%)	20 243 (60.9%)
Heterosexual sex	19 745 (33.1%)	10 258 (38.7%)	9487 (28.5%)
Injecting drug use	5485 (9.2%)	3551 (13.4%)	1934 (5.8%)
Transfusion, other, or unknown	3224 (5.3%)	1640 (6.2%)	1584 (4.8%)
Missing	0	0	0
Race/ethnicity			
White	45 666 (76.4%)	19 836 (74.9%)	25 830 (77.7%)
Black	7787 (13.0%)	4257 (16.1%)	3530 (10.6%)
Hispanic	3114 (5.2%)	997 (3.8%)	2117 (6.4%)
Other	2351 (4.0%)	921 (3.4%)	1430 (4.3%)
Unknown	823 (1.4%)	482 (1.8%)	341 (1.0%)
Missing	0	0	0
Viral load (copies/mL)			
<100	52 294 (90.2%)	22 317 (85.5%)	29 977 (94.0%)
101–1000	2271 (3.9%)	1352 (5.2%)	919 (2.9%)
1001–10 000	1147 (2.0%)	803 (3.1%)	344 (1.1%)
10 001	2244 (3.9%)	1606 (6.2%)	638 (2.0%)
Missing	1785	415	1370
Fibrosis-4 index			
<1.45	39 449 (83.2%)	15 618 (78.9%)	39 449 (83.2%)
1.45–3.25	7069 (14.9%)	3577 (18.1%)	7069 (14.9%)
>3.25	917 (1.9%)	595 (3.0%)	917 (1.9%)
Missing	12 306	6703	5603
Continuous characteristics (mean)			
Age in years	43	44	42
Missing	0	0	0
Albumin (g/dL)	4.3	4.2	4.3
Missing	32 875	14 551	18 324
Hemoglobin (g/dL)	13.7	13.5	13.9
Missing	7405	4287	3118
White blood cell (10^3^/µL)	6.3	6.2	6.4
Missing	16 816	7154	9662
CD4 count (cells/mm^3^)	605.2	545.7	655.0
Missing	20 772	8746	12 026
Estimated glomerular filtration rate (mL/min/1.73 m²)	137.1	142.5	132.7
Missing	1773	1063	710
Body mass index (kg/m^3^)	24.8	24.8	24.8
Missing	10 885	4015	6870

Abbreviation: ART, antiretroviral therapy.

More than half (33 248 [55.7%]) of the included PWH started ART between 2010 and 2018. PWH who started follow-up in 2001 had been on ART for a median of 1.2 (IQR: 1.1–1.4) years beforehand, whereas the median prior duration on ART was 3.3 (IQR: 1.7–6.1) years for PWH who started follow-up in 2015. The median CD4 counts at baseline increased from 216 (IQR: 64–359) for those starting follow-up in 2001 to 345 (IQR: 190–504) cells/mm^3^ for those starting in 2018. Compared to PWH who started ART between 2000 and 2009, a lower proportion of those who initiated ART between 2010 and 2018 had AIDS at the time of initiation (12.8% [4251] versus 24.3% [6428]), viral loads above 1000 copies/mL (3.1% [982] vs 9.3% [2409]), and hepatitis C virus (5.8% [1920] vs 12.4% [3273]). Overall, 9927 (16.6%) PWH had complete records for all laboratory components of the VACS Index 2.0. Missingness ranged from 1773 (3.0%) PWH without creatinine data to 32 875 (55.0%) PWH without albumin data.

### All-cause Mortality

Of the 59 741 PWH, 2425 (4.1%) died over 5 years of follow-up. The mean age at death was 51.2 (95% confidence interval [CI], 50.8–51.6) years. The 5-year mortality rate decreased from 64.1/1000 person-years for PWH who started in 2001 to 8.2/1000 person-years for people starting follow-up in 2015. Using imputed data, among PWH who did not subsequently die, the mean VACS Index 2.0 was 41.2 (95% CI, 41.1–41.4) and was 45.4 (95% CI, 45.1–45.6) for PWH who started in 2000–2009, and 38.1 (95% CI, 37.9–38.3) in 2010–2018. Among PWH who subsequently died, the mean VACS Index 2.0 was 68 (95% CI, 67–68) in 2000–2018, 69 (95% CI, 68–70) in 2000–2009, and 63 (95% CI, 62–65) in 2010–2018 (Table 2). The estimated all-cause mortality HR per 10-unit increase in VACS Index 2.0 was 1.84 (95% CI, 1.81–1.87), and the corresponding C-statistic for discrimination was 0.83 (Table 3).

**Table 2.** VACS Index 2.0 at Follow-up Start by Cause of Death, Using Multiply Imputed Data

Cause Of Death	Year Of ART Start					
	2000–2018		2000–2009		2010–2018	
	No. of deaths	Mean (95% CI) VACS Index 2.0	No. of deaths	Mean (95% CI) VACS Index 2.0	No. of deaths	Mean (95% CI) VACS Index 2.0
All-cause	2425	68 (67–68)	1887	69 (68–70)	538	63 (62–65)
AIDS	455	78 (76–80)	363	79 (76–81)	92	74 (69–78)
Liver (including HCC)	148	76 (72–80)	122	77 (73–81)	26	70 (60–80)
Cardiovascular	214	62 (59–65)	166	63 (60–66)	48	58 (53–63)
Respiratory	99	72 (68–75)	75	70 (66–74)	24	76 (66–86)
Non-AIDS infection	141	72 (68–76)	109	73 (69–78)	32	66 (58–74)
Non–AIDS-defining cancers	452	66 (64–68)	351	67 (64–69)	101	65 (61–69)
Other	306	66 (63–69)	256	67 (64–70)	50	61 (55–67)
Substance abuse	97	62 (58–66)	76	62 (57–67)	21	61 (50–73)
Suicide/accident	141	50 (46–54)	98	51 (47–55)	43	48 (42–54)
Unclassifiable	372	63 (61–65)	271	65 (63–68)	101	58 (53–62)

Abbreviations: ART, antiretroviral therapy; CI, confidence interval; HCC, hepatocellular carcinoma; VACS, Veterans Aging Cohort Study.

**Table 3.** All-cause and Cause-specific Mortality Hazard Ratios per 10-point Increment of VACS Index 2.0 and Discrimination C-statistic, Using Multiply Imputed Data

Cause of Death	2000–2018		2000–2009		2010–2018	
	Hazard ratio (95% CI)	C-statistic	Hazard ratio (95% CI)	C-statistic	Hazard ratio (95% CI)	C-statistic
All-cause	1.84 (1.81–1.87)	0.83	1.77 (1.73–1.80)	0.81	1.97 (1.90–2.05)	0.83
AIDS	2.14 (2.06–2.23)	0.91	2.06 (1.97–2.16)	0.89	2.27 (2.09–2.47)	0.93
Liver (including HCC)	2.13 (1.99–2.29)	0.91	2.08 (1.92–2.25)	0.89	2.26 (1.92–2.66)	0.90
Cardiovascular	1.66 (1.56–1.77)	0.79	1.59 (1.48–1.71)	0.76	1.82 (1.59–2.10)	0.79
Respiratory	2.00 (1.83–2.18)	0.89	1.84 (1.66–2.05)	0.86	2.42 (2.05–2.86)	0.94
Non-AIDS infection	1.98 (1.85–2.13)	0.87	1.93 (1.77–2.09)	0.85	2.04 (1.76–2.35)	0.83
Non–AIDS-defining cancers	1.81 (1.73–1.89)	0.83	1.69 (1.61–1.78)	0.79	2.04 (1.87–2.23)	0.87
Other	1.76 (1.67–1.85)	0.78	1.69 (1.60–1.79)	0.76	1.85 (1.63–2.10)	0.79
Substance abuse	1.70 (1.56–1.87)	0.83	1.60 (1.44–1.77)	0.81	1.92 (1.58–2.34)	0.82
Suicide/accident	1.32 (1.20–1.44)	0.65	1.25 (1.12–1.39)	0.63	1.41 (1.19–1.68)	0.69
Unclassifiable	1.70 (1.62–1.78)	0.77	1.66 (1.57–1.76)	0.77	1.74 (1.58–1.91)	0.75

Abbreviations: CI, confidence interval; HCC, hepatocellular carcinoma; VACS, Veterans Aging Cohort Study.

**Figure 1.**
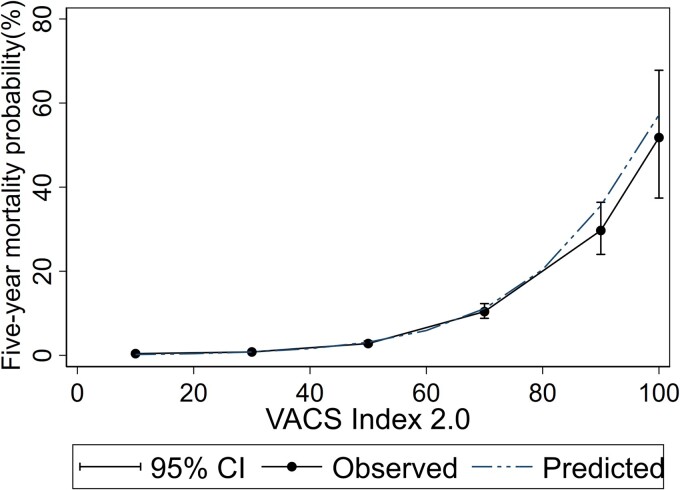
Five-year probability of predicted and observed all-cause mortality.

The predicted 5-year mortality for PWH with a mean VACS Index 2.0 score of 38 at baseline during 2010–2018 was 1.3%. The 5-year observed and predicted risks of dying were similar in PWH who started ART in 2010–2018. However, the absolute risk of dying fitted better for lower scores of the VACS Index 2.0 than for higher values, where there were fewer observations (Figure 1). The predicted risk of all-cause mortality approximately doubled at every 10-unit interval of the VACS Index 2.0 (Table 4).

**Table 4.** Five-year Predicted all-cause and Cause-specific Mortality Percentages by VACS Index 2.0 Score, Using Multiply Imputed Data

	VACS Index 2.0									
	10	20	30	40	50	60	70	80	90	100
All-cause	0.2%	0.4%	0.8%	1.6%	3.1%	5.9%	11.1%	20.4%	35.6%	57.2%
AIDS	0.0%	0.0%	0.1%	0.1%	0.3%	0.7%	1.5%	3.4%	7.6%	16.5%
Liver (including HCC)	0.0%	0.0%	0.0%	0.1%	0.1%	0.3%	0.7%	1.4%	3.1%	6.7%
Cardiovascular	0.0%	0.1%	0.1%	0.2%	0.4%	0.6%	1.1%	1.9%	3.3%	5.6%
Respiratory	0.0%	0.0%	0.0%	0.0%	0.1%	0.2%	0.5%	1.2%	2.8%	6.5%
Non-AIDS infection	0.0%	0.0%	0.0%	0.1%	0.2%	0.3%	0.6%	1.3%	2.5%	5.1%
Non–AIDS-defining cancers	0.0%	0.1%	0.2%	0.3%	0.6%	1.2%	2.4%	4.6%	9.0%	17.1%
Other	0.0%	0.0%	0.1%	0.2%	0.3%	0.6%	1.0%	1.9%	3.5%	6.3%
Substance abuse	0.0%	0.0%	0.0%	0.1%	0.2%	0.3%	0.5%	1.0%	1.9%	3.4%
Suicide/accident	0.1%	0.1%	0.2%	0.2%	0.3%	0.4%	0.5%	0.7%	1.0%	1.3%
Unclassifiable	0.1%	0.1%	0.3%	0.4%	0.7%	1.3%	2.2%	3.7%	6.2%	10.4%

Abbreviations: HCC, hepatocellular carcinoma; VACS, Veterans Aging Cohort Study..

### Cause-specific Mortality

The 5 most frequent causes of death were AIDS (n = 455), NADCs (n = 452), cardiovascular diseases (n = 214), liver-related diseases (n = 148), and non-AIDS infection (n = 141). Other causes of death were suicide/accident (n = 141), respiratory (n = 99), and substance abuse (n = 97) (Table 2). For PWH who started ART during 2000–2009, the mean VACS Index 2.0 was highest for PWH who subsequently died of AIDS (79 [95% CI, 76–81]), and lowest for suicide/accidental deaths (51 [95% CI, 47–55]). For PWH who started ART during 2010–2018, the mean VACS Index 2.0 was highest for those who subsequently died of respiratory causes (76 [95% CI, 66–86]) and AIDS (74 [95% CI, 69–78]), whereas PWH who subsequently died from suicide/accidents had the lowest mean VACS Index 2.0 (48 [95% CI, 42–54]).

For PWH who started ART between 2000 and 2009, discrimination of the VACS Index 2.0 was highest for AIDS and liver-related deaths (0.89), followed by respiratory-related deaths (0.86), and was lowest for suicides/accidental deaths (0.63) (Table 3). For PWH who started ART from 2010 to 2018, discrimination was highest for deaths from respiratory infections (0.94), AIDS (0.93), liver diseases (0.90), NADCs (0.87), and non-AIDS infections (0.83). Again, suicide/accident-related deaths had the lowest discrimination (0.69). The highest C-statistics corresponded to the highest HRs, for example the highest HR was for AIDS (2.27 [95% CI, 2.09–2.47]) and the lowest was for suicide/accidental deaths (1.25 [95% CI, 1.12–1.39]). Compared to the predicted risk of deaths from NADCs and AIDS, which roughly doubled per 10-unit intervals of the VACS Index 2.0, suicide/accidents deaths only increased slightly as the VACS Index 2.0 increased (Table 4).

### Sensitivity Analyses

Most characteristics were similar among PWH with and without complete data, except for cohort (Supplementary Table 4). After adjusting for confounding, PWH who had died were more likely to have missing data than those who did not die, as were those who started ART in 2000–2009 compared with those starting ART in 2010–2018 (Supplementary Table 5). Sensitivity analysis using complete case data was similar to the main results from imputed data, showing that AIDS, liver-, and respiratory-related deaths had the highest discrimination, whereas deaths from suicide/accidents had the lowest discrimination. The mean VACS Index 2.0 at baseline was similar in the complete-case and imputed analysis (Supplementary Table 6). In complete-case analysis (N = 9927), the mean VACS Index 2.0 for people who did not and did die was 39.9 (95% CI, 39.5–40.3) (n = 9490) and 68 (95% CI, 66–70) (n = 437), respectively.

All-cause and cause-specific HRs were similar when using Cox models and competing risk models (Supplementary Table 7). However, the C-statistics differed for substance abuse deaths, 0.74 (95% CI, .65–.84) and 0.89 (95% CI, .84–.94) using Cox models and competing risk models, respectively. This was also the case for suicide/accidental deaths, 0.51 (95% CI, .39–.62) and 0.73 (95% CI, .65–.82), and unclassifiable deaths, 0.81 (95% CI, .74–.87) and 0.89 (95% CI, .84–.93).

In subgroup analysis, the mean VACS Index 2.0 scores and C-statistics were similar in both men and women for all-cause and cause-specific mortality, except for respiratory, non-AIDS infection, and suicide/accident-related deaths (Supplementary Table 8). The mean VACS Index 2.0 score for all-cause mortality was lower in people with White ethnicity than non-White ethnicity. By different causes of death, the C-statistics differed for deaths from liver, respiratory, non-AIDS infection, and substance abuse (Supplementary Table 9).

## DISCUSSION

The VACS Index 2.0 demonstrated better predictive 5-year accuracy for deaths attributed to AIDS, liver disease, and respiratory infections than for suicides or accidental deaths. As expected, discrimination was lowest for suicides or accidental deaths, for which the predictors were not included in the calculation of the VACS Index 2.0. The VACS Index 2.0 showed better discrimination of most causes of death among PWH who started ART between 2010 and 2018, compared to those who started ART between 2000 and 2009, particularly for NADC deaths. The 5-year probability of mortality increases nearly twofold with every 10-unit increase of the VACS Index 2.0.

The discrimination of the VACS Index 2.0 in predicting 5-year all-cause mortality in our study, 0.83, was the same as in a previous study by Tate et al [8]. That study also found that the risk of all-cause mortality increased for PWH with higher VACS Index 2.0 scores. Better performance of the VACS Index 2.0 in more recent years (2010–2018) compared to earlier years (1999–2009) was reported in a study of 4 clinical cohorts in North America [10], similar to our results. This could be due to the original calibration and validation of the VACS Index 2.0 taking place during the 2010–2018 period, aligning the model with more current data and trends.

The major strength of this study is the coding of cause-specific mortality among a large collaboration of HIV cohorts across many high-income countries, encompassing diverse demographics and clinical profiles. Use of standardized CoDe protocol methods further strengthens the study's results by improving the accuracy in determining the cause of death among PWH. However, there is a possibility that for some PWH, causes of death were misclassified because of retrospective assignment without complete patient history information. Additionally, there may have been underascertainment of mortality because 6 of 12 ART-CC cohorts were not linked to vital statistics registries. However, these cohorts employed alternative methods to identify deaths among PWH who were lost to follow-up, such as linking with healthcare providers and family members. A substantial proportion (83%) of PWH had missing data on laboratory components of the VACS Index 2.0. This missingness could be attributed to differences in data collection methods across the cohorts, resulting from variations in HIV care protocols. Persons that had died were also more likely to have missing data, perhaps because suboptimal adherence to HIV clinic visits (in which data would have been collected) is associated with increased risk of death [29]. Despite this limitation, results were similar for complete case and imputed analyses. Last, because of computational issues for the combination of multiple imputation and competing risks methods, we were unable to account for competing risks of mortality in the main analyses. However, in complete case analyses, the associations between the VACS Index 2.0 and cause-specific mortality were similar under a standard framework and a competing risks framework.

The high discrimination of the VACS Index 2.0 for all-cause and cause-specific mortality suggests that the model can be useful to predict deaths, particularly those caused by AIDS, and liver disease. This can be attributed to the inclusion of markers associated with these conditions, such as the Fibrosis-4 score for liver disease and CD4 count and viral load for AIDS. However, the VACS Index 2.0 performed less well at discriminating cardiovascular-related deaths. To improve discrimination for this cause, it may be necessary to update the VACS Index 2.0 to include additional biomarkers such as lipid profiles that can serve as indicators of cardiovascular health [30]. Although it is possible that inclusion of mental health components to the VACS Index 2.0 could improve prediction of suicide/accidental death, the VACS Index 2.0 was designed to assess physiological frailty only [12]. The limited predictive capability of the VACS Index 2.0 for suicide serves as a validation of its intended purpose.

Nevertheless, together with clinical judgment, the VACS Index 2.0 can provide information for PWH about the risk of dying from the most common causes. This information is crucial for designing targeted interventions and tailoring care plans to address the specific risks identified by the VACS Index 2.0. For instance, if the index highlights a high risk of NADC death, lifestyle modifications or further monitoring and testing may be recommended. Alternatively, the VACS Index 2.0 can also help identify PWH who are at low risk of dying. By identifying those at low risk, healthcare resources can be directed toward individuals with higher risk profiles, ensuring that interventions and care are focused where they are most needed. The availability of VACS Index 2.0 information can serve as a motivator for PWH to adopt healthier habits and improve adherence to ART.

## Supplementary Material

ofae333_Supplementary_Data

## Supplementary Data

Supplementary materials are available at *Open Forum Infectious Diseases* online. Consisting of data provided by the authors to benefit the reader, the posted materials are not copyedited and are the sole responsibility of the authors, so questions or comments should be addressed to the corresponding author.

## Notes

***Acknowledgments***. We thank our funders (US NIAAA) as well as all the patients and the clinical teams associated with the participating cohort studies.

***Author Contributions*.** A.J., A.T., S.I., and J.A.C.S. conceived and designed the study. A.T., L.Z., and S.I. combined, checked, and cleaned the datasets. M.J.G., S.A., J.B., C.W., M.H., and P.R. were involved in the cross-cohort coding of causes of death. J.A. conducted the statistical analyses. J.A. drafted the manuscript. All authors contributed to the interpretation of data and critical revisions of the manuscript for important intellectual content. A.T., S.I., J.A., and J.A.C.S. had full access to all the data in the study. J.A., S.I., A.T., and J.A.C.S. were responsible for the decision to submit for publication.

***Data sharing statement*.** Due to the data sharing agreements between individual cohorts and ART-CC, the data collected for this study cannot be shared. Data are owned by the individual cohorts and those wishing to access these data should contact the individual cohorts.

***Patient consent statement*.** Ethics committees or institutional review boards approved the 12 individual cohorts, each of which used standardized data collection methods and conducted regular follow-up with participants who had consented to be included. Additionally, the ART-CC as a whole received ethics approval from the UK NHS National Research Ethics Service Committee South West—Cornwall & Plymouth (12/SW/0253).

***Financial support*.** The ART-CC is funded by the US National Institute on Alcohol Abuse and Alcoholism (U01-AA026209). Funding for the individual ART-CC cohorts was from Alberta Health, Gilead, ANRS (France REcherche Nord&Sud Sida-hiv Hépatites), the French Ministry of Health, the Austrian Agency for Health and Food Safety (AGES), Stichting HIV Monitoring, the Dutch Ministry of Health, Welfare and Sport through the Centre for Infectious Disease Control of the National Institute for Public Health and the Environment, the TP-HIV by the German Centre for Infection Research (DZIF) (NCT02149004), the Instituto de Salud Carlos III through the Red Temática de Investigación Cooperativa en Sida (RD06/006, RD12/0017/0018, and RD16/0002/0006) as part of the Plan Nacional I + D + i and cofinanced by ISCIII-Subdirección General de Evaluación and the Fondo Europeo de Desarrollo Regional (FEDER), ViiV Healthcare, Preben og Anna Simonsens Fond, ANRS-Maladies infectieuses émergentes, Institut National de la Santé et de la Recherche Médicale (INSERM), BMS, Janssen, MSD, the US National Institute on Alcohol Abuse and Alcoholism (U01-AA026230), the Spanish Ministry of Health, the Swiss National Science Foundation (grant 33CS30_134277), CFAR Network of Integrated Clinical Systems (1R24 AI067039-1, P30-AI-027757), the US Department of Veterans Affairs, the US National Institute on Alcohol Abuse and Alcoholism (U01-AA026224, U01-AA026209, U24-AA020794), the VHA Office of Research and Development, US National Institute of Allergy and Infectious Diseases (Tennessee Center for AIDS Research: P30 AI110527).
