# Supplementary material for: Discrimination of the Veterans Aging Cohort Study Index 2.0 for Predicting Cause-specific Mortality Among Persons With HIV in Europe and North America
Source: Open Forum Infect Dis. 2024 Jun 17;11(7):ofae333. doi: 10.1093/ofid/ofae333 (PMC11249920; doi:10.1093/ofid/ofae333)
Supplement: ofae333_Supplementary_Data [file ofae333_supplementary_data.docx]

**Discrimination of the Veterans Aging Cohort Study Index 2.0 for predicting cause-specific mortality among persons with HIV in Europe and North America**

**Appendix**

**Contents**

[Cause of death categories 2](#_Toc162878759)

[Knots for the Royston and Parmar model 4](#_Toc162878760)

[Assessing linear and non-linear association of VACS Index 2.0 and cause-specific mortality using Bayesian Information Criterion 7](#_Toc162878761)

[Analysis done using complete cases. 10](#_Toc162878762)

[Predictors of missingness 11](#_Toc162878763)

[Multiple imputations 14](#_Toc162878764)

[Compatibility between the imputation model and the complete case model 14](#_Toc162878765)

[References 17](#_Toc162878766)

# **Cause of death categories**

Ten main categories of causes of death were included in the analysis (Supplementary Table 1). Causes of death that had less than 100 events were grouped as ‘other’. Hepatocellular carcinoma was counted with liver disease.

**Supplementary Table 1. Grouped causes of death and corresponding Coding of Death in HIV protocol codes**

| **Grouped causes of death** | **Coding of Death in HIV protocol codes** |
| --- | --- |
| AIDS | 01 AIDS |
|  | 01.1 Infection |
|  | 01.2 Malignancy |
| Non-AIDS infection | 02 Infection (excluding 01.1)  02.1 Bacterial  02.1.1 Bacterial with sepsis  02.2 Others  02.2.1 Other with sepsis  02.3 Unknown aetiology  02.3.1 Unknown with sepsis |
| Non-AIDS defining cancers | 04 Malignancy |
| Liver | 03 Chronic viral hepatitis |
|  | 03.1 Hepatitis C virus (HCV) |
|  | 03.1.1 HCV with cirrhosis |
|  | 03.1.2 HCV with liver failure |
|  | 03.1.3 HCV with liver cancer |
|  | 03.2 Hepatitis B virus (HBV) |
|  | 03.2.1 HBV with cirrhosis |
|  | 03.2.2 HBV with liver failure |
|  | 03.2.3 HBV with liver cancer |
|  | 04.2 Liver cancer |
| Cardiovascular | 08.1 Acute myocardial infarction (AMI) |
|  | 08.1.1 Definitive AMI |
|  | 08.1.2 Possible AMI |
|  | 08.2 Other ischemic heart disease |
|  | 09 Stroke |
|  | 24 Heart or vascular (other causes) |
| Respiratory | 13 Chronic obstructive lung disease |
|  | 25 Respiratory disease (other causes) |
| Substance abuse | 19 Substance abuse (active) |
|  | 19.1 Chronic alcohol abuse |
|  | 19.2 Chronic intravenous drug-use |
|  | 19.3 Acute intoxication |
| Unclassifiable | 91 Unclassifiable causes |
|  | 92 Unknown |
|  | 92.1 Unknown, competing risks |
| Other | 23 Central nervous system disease |
|  | 90 Other causes |
| Suicide/accident | 16 Accident or other violent death |
|  | 17 Suicide |
|  | 18 Euthanasia |

# **Knots for the Royston and Parmar model**

Selection of the number of interior knots to use when modelling the baseline cumulative hazard (1-4) was made based on the lowest values of BIC (underlined numbers) (Supplementary Table 2). Based on this assessment, it was determined that a Royston and Parmar model with 2 degrees of freedom, corresponding to one internal knot positioned at the 50th percentile, provided a satisfactory fit to the data for all outcomes of interest.

**Supplementary Table 2. Assessing degree of freedom to model baseline hazard using Bayesian Information Criterion**

| **Outcome** | **Degrees of freedom** | **BIC** |
| --- | --- | --- |
| All-cause | 2 | 6951.025 |
|  | 3 | 6961.294 |
|  | 4 | 6965.115 |
|  | 5 | 6975.590 |
| AIDS | 2 | 1586.463 |
|  | 3 | 1596.511 |
|  | 4 | 1602.226 |
|  | 5 | 1611.176 |
| Liver | 2 | 530.282 |
|  | 3 | 540.582 |
|  | 4 | 550.975 |
|  | 5 | 561.036 |
| Cardiovascular | 2 | 850.587 |
|  | 3 | 856.951 |
|  | 4 | 866.512 |
|  | 5 | 877.157 |
| Respiratory | 2 | 489.200 |
|  | 3 | 492.780 |
|  | 4 | 502.802 |
|  | 5 | 512.724 |
| Non-AIDS infection | 2 | 636.611 |
|  | 3 | 644.553 |
|  | 4 | 654.170 |
|  | 5 | 664.108 |
| Non-AIDS defining | 2 | 1705.081 |
|  | 3 | 1713.127 |
|  | 4 | 1722.565 |
|  | 5 | 1732.758 |
| Other | 2 | 922.323 |
|  | 3 | 932.735 |
|  | 4 | 942.731 |
|  | 5 | 953.275 |
| Substance abuse | 2 | 429.489 |
|  | 3 | 439.215 |
|  | 4 | 448.802 |
|  | 5 | 452.948 |
| Unclassifiable | 2 | 1628.748 |
|  | 3 | 1636.040 |
|  | 4 | 1645.925 |
|  | 5 | 1655.156 |
| Suicide/accidents | 2 | 757.887 |
|  | 3 | 768.033 |
|  | 4 | 777.483 |
|  | 5 | 786.730 |

Analysis done using complete cases.

#

# **Assessing linear and non-linear association of VACS Index 2.0 and cause-specific mortality using Bayesian Information Criterion**

The association between the outcome (cause-specific mortality) and continuous exposure (VACS Index 2.0) was assessed using complete cases. Linearity was examined using Royston-Parmar flexible parametric survival model. Non-linear associations of the VACS Index 2.0 were modelled using decile, quintile and dichotomous categories of the VACS Index 2.0, and the linear association was modelled using VACS Index 2.0 as continuous. VACS Index 2.0 modelled as decile showed that PWH with higher VACS Index 2.0 scores had an increased risk of dying compared to the PWH who had VACS Index 2.0 of 0-19 (Supplementary Figure 1). Similarly, VACS Index 2.0 grouped into 10-unit intervals showed an increasing hazard ratio with increasing VACS Index 2.0. Overall, VACS Index 2.0 as continuous fitted the data better than the non-linear forms (Supplementary Table 3).

**Supplementary Figure 1. All-cause mortality hazard ratio in deciles of the VACS Index 2.0**

Analysis done using complete cases.

**Supplementary Table 3. Assessing linear and non-linear association of VACS Index 2.0 and cause-specific mortality using Bayesian Information Criterion**

|  | **VACS Index 2.0 as continuous** | **VACS Index 2.0 divided into 2 equal sample-size groups (dichotomous)** | **VACS Index 2.0 divided into 5 equal sample-size groups (quintile)** | **VACS Index 2.0 divided into 10 equal sample-size groups (decile)** |
| --- | --- | --- | --- | --- |
| All-cause | 1114.820 | 1264.398 | 1206.533 | 1202.799 |
| AIDS | 368.161 | 424.762 | 421.624 | 445.556 |
| Liver | 107.836 | 119.213 | 135.219 | Lack of  convergence |
| Cardiovascular | 191.487 | 194.429 | Lack of  convergence | Lack of  convergence |
| Respiratory | Lack of  convergence | 71.755 | Lack of  convergence | Lack of  convergence |
| Non-AIDS infection | 155.632 | 168.970 | 185.405 | 225.588 |
| Non-AIDS defining cancers | 314.135 | 345.241 | 346.458 | 378.690 |
| Other | 126.428 | 142.035 | 156.322 | 190.642 |
| Substance abuse | 139.024 | 143.084 | 160.625 | 200.126 |
| Suicide/accident | 135.485 | 135.779 | 154.201 | 192.078 |
| Unclassifiable | 231.338 | 241.505 | 260.642 | 287.254 |

Analysis done using complete cases.

**Comparison between persons with HIV with complete and incomplete data**

In univariable analysis, characteristics of PWH with and without complete data on the 10 variables of the VACS Index 2.0 components were similar (Supplementary Table 4). However, differences were observed in the percentage of PWH with complete data and those with incomplete data that were from each cohort and among persons who acquired HIV through injecting drug use.

**Supplementary Table 4. Comparison of persons with HIV with complete VACS Index 2.0 components and those with missing data**

|  | **Total** | **Complete data on all VACS Index 2.0 components** | **Missing data on ≥1 VACS Index 2.0 component** |
| --- | --- | --- | --- |
|  | 59,741 (100.0%) | 9,927 (16.6%) | 49,814 (83.4%) |
| **Dead** |  |  |  |
| No | 57,316 (95.9%) | 9,490 (95.6%) | 47,826 (96.0%) |
| Yes | 2,425 (4.1%) | 437 (4.4%) | 1,988 (4.0%) |
| **Age in years, mean** | 43 | 43 | 43 |
| **Sex** |  |  |  |
| Male | 47,842 (80.1%) | 7,667 (77.2%) | 40,175 (80.7%) |
| Female | 11,899 (19.9%) | 2,260 (22.8%) | 9,639 (19.3%) |
| **Probable route of HIV acquisition** |  |  |  |
| Men who have sex with men | 31,287 (52.4%) | 4,591 (46.2%) | 26,696 (53.6%) |
| Injecting drug use | 5,485 (9.2%) | 1,386 (14.0%) | 4,099 (8.2%) |
| Heterosexual sex | 19,745 (33.1%) | 3,464 (34.9%) | 16,281 (32.7%) |
| Transfusion, other, or unknown | 3,224 (5.4%) | 486 (4.9%) | 2,738 (5.5%) |
| **Ethnic** |  |  |  |
| White | 45,666 (76.4%) | 7,292 (73.5%) | 38,374 (77.0%) |
| Black | 7,787 (13.0%) | 1,637 (16.5%) | 6,150 (12.3%) |
| Hispanic | 3,114 (5.2%) | 341 (3.4%) | 2,773 (5.6%) |
| Other | 2,351 (3.9%) | 459 (4.6%) | 1,892 (3.8%) |
| Unknown | 823 (1.4%) | 198 (2.0%) | 625 (1.3%) |
| **AIDS at ART initiation** |  |  |  |
| No | 49,062 (82.1%) | 8,099 (81.6%) | 40,963 (82.2%) |
| Yes | 10,679 (17.9%) | 1,828 (18.4%) | 8,851 (17.8%) |
| **Laboratory values** |  |  |  |
| Aspartate aminotransferase (U/L), mean | 28 | 28 | 28 |
| Albumin (g/dL) mean | 4.3 | 4.2 | 4.3 |
| Alanine aminotransferase (U/L), mean | 29 | 29 | 29 |
| Creatinine (mg/dL), mean | 0.9 | 0.9 | 0.9 |
| Haemoglobin (g/dL), mean | 13.7 | 14.0 | 13.6 |
| Platelets (10^9^/L), mean | 230 | 226 | 231 |
| White blood cells (10^3^/µL), mean | 6.3 | 6.3 | 6.3 |
| CD4 at ART initiation (cells/mm^3^), mean | 605 | 580 | 614 |
| Viral load at ART initiation (expressed as log 10 copies/mL, mean) | 0.6 | 0.6 | 0.5 |
| **Hepatitis C virus RNA** |  |  |  |
| No | 54,548 (91.3%) | 8,875 (89.4%) | 45,673 (91.7%) |
| Yes | 5,193 (8.7%) | 1,052 (10.6%) | 4,141 (8.3%) |
| Weight (kg), mean | 74.9 | 75.2 | 74.8 |
| Height (m), mean | 1.7 | 1.7 | 1.7 |
| **Calendar period** |  |  |  |
| 2000-2009 | 26,493 (44.3%) | 4,833 (48.7%) | 21,660 (43.5%) |
| 2010-2018 | 33,248 (55.7%) | 5,094 (51.3%) | 28,154 (56.5%) |
| **Cohort** |  |  |  |
| A | 3,674 (6.1%) | 1,340 (13.5%) | 2,334 (4.7%) |
| B | 3,511 (5.9%) | 216 (2.2%) | 3,295 (6.6%) |
| C | 14,119 (23.6%) | 1,008 (10.2%) | 13,111 (26.3%) |
| D | 1,169 (2.0%) | 919 (9.3%) | 250 (0.5%) |
| E | 1,810 (3.0%) | 169 (1.7%) | 1,641 (3.3%) |
| F | 8,930 (14.9%) | 0 (0.0%) | 8,930 (17.9%) |
| G | 8,317 (13.9%) | 945 (9.5%) | 7,372 (14.8%) |
| H | 1,036 (1.7%) | 64 (0.6%) | 972 (2.0%) |
| I | 6,358 (10.6%) | 983 (9.9%) | 5,375 (10.8%) |
| J | 1,502 (2.5%) | 1,102 (11.1%) | 400 (0.8%) |
| K | 7,228 (12.1%) | 2,390 (24.1%) | 4,838 (9.7%) |
| L | 2,087 (3.5%) | 791 (8.0%) | 1,296 (2.6%) |

# Analysis done using complete cases.

# **Predictors of missingness**

Ten variables that had no missing data (n = 59,741) were fitted in a logistic regression model (Supplementary Table 5). However, the model failed to converge due to quasi-complete separation. In cohort F, all PWH were without documented white blood cells values. Therefore, Firth's penalised maximum likelihood method(1) was implemented using Stata’s FIRTH command. The following confounders were adjusted for in the multivariable model: age, sex, ethnicity, mode of HIV acquisition, presence of AIDS at ART initiation, hepatitis C infection, viral load at ART initiation, year of starting ART, and cohort. In multivariable analysis, the adjusted odds of having incomplete records were 31% higher (OR: 1.31; 95% CI 1.16-1.49) among persons who had died than those who were alive. Compared to PWH who started ART between 2010 and 2018, those who started ART in 2000-2009 were more likely to have missing data (OR: 1.22; 95% CI 1.16-1.29).

**Supplementary Table 5. Predictors of having incomplete records**

|  | **Unadjusted odds ratio (95% CI)** | **p- value** | **Adjusted odds ratio (95% CI)** | **p- value** |
| --- | --- | --- | --- | --- |
| **Dead** |  |  |  |  |
| No | 1.00 |  | 1.00 |  |
| Yes | 0.90 (0.81-1.00) | 0.058 | 1.31 (1.16-1.49) | <0.001 |
| **Age in years** |  |  |  |  |
| <20 | 0.75 (0.47-1.21) | 0.236 | 1.18 (0.68-2.03) | 0.563 |
| 20-24 | 0.86 (0.75-1.00) | 0.045 | 1.03 (0.87-1.21) | 0.729 |
| 25-29 | 0.89 (0.80-0.98) | 0.024 | 1.08 (0.95-1.22) | 0.226 |
| 30-34 | 1.05 (0.95-1.15) | 0.355 | 1.13 (1.01-1.26) | 0.033 |
| 35-39 | 1.05 (0.95-1.15) | 0.360 | 1.16 (1.04-1.29) | 0.006 |
| 40-44 | 1.01 (0.92-1.11) | 0.884 | 1.16 (1.05-1.29) | 0.005 |
| 45-49 | 0.92 (0.84-1.01) | 0.098 | 1.09 (0.98-1.22) | 0.094 |
| 50-54 | 0.87 (0.79-0.96) | 0.007 | 1.06 (0.95-1.18) | 0.336 |
| 55-59 | 0.92 (0.82-1.02) | 0.119 | 1.02 (0.90-1.15) | 0.785 |
| ≥60 | 1.00 |  | 1.00 |  |
| **Sex** |  |  |  |  |
| Female | 1.00 |  | 1.00 |  |
| Male | 1.23 (1.17-1.29) | <0.001 | 1.13 (1.05-1.21) | 0.001 |
| **Ethnic** |  |  |  |  |
| White | 1.00 |  | 1.00 |  |
| Black | 0.71 (0.67-0.76) | <0.001 | 0.87 (0.81-0.94) | 0.001 |
| Hispanic | 1.55 (1.38-1.73) | <0.001 | 1.08 (0.93-1.26) | 0.321 |
| Other | 0.78 (0.71-0.87) | <0.001 | 1.06 (0.93-1.21) | 0.378 |
| Unknown | 0.60 (0.51-0.71) | <0.001 | 0.85 (0.71-1.02) | 0.074 |
| **Probable route of HIV acquisition** |  |  |  |  |
| Men who have sex with men | 1.00 |  | 1.00 |  |
| Injecting drug use | 0.51 (0.47-0.54) | <0.001 | 1.03 (0.93-1.13) | 0.602 |
| Heterosexual sex | 0.81 (0.77-0.85) | <0.001 | 1.01 (0.94-1.08) | 0.786 |
| Transfusion, other, or unknown | 0.97 (0.88-1.07) | 0.541 | 0.87 (0.78-0.97) | 0.015 |
| **AIDS at ART initiation** |  |  |  |  |
| No | 1.00 |  | 1.00 |  |
| Yes | 0.96 (0.91-1.01) | 0.125 | 0.95 (0.89-1.01) | 0.093 |
| **Hepatitis C infection** |  |  |  |  |
| No | 1.00 |  | 1.00 |  |
| Yes | 0.76 (0.71-0.82) | <0.001 | 0.89 (0.81-0.99) | 0.027 |
| **Viral load at ART initiation** |  |  |  |  |
| <101 | 0.61 (0.48-0.79) | <0.001 | 0.81 (0.62-1.07) | 0.146 |
| 101-1000 | 0.76 (0.67-0.86) | <0.001 | 0.90 (0.78-1.04) | 0.172 |
| 1001-10000 | 1.00 (0.93-1.07) | 0.905 | 1.12 (1.04-1.21) | 0.005 |
| ≥10000 | 1.00 |  | 1.00 |  |
| **Calendar period at ART initiation** |  |  |  |  |
| 2000-2009 | 0.81 (0.78-0.85) | <0.001 | 1.22 (1.16-1.29) | <0.001 |
| 2010-2018 | 1.00 |  | 1.00 |  |
| **Cohort** |  |  |  |  |
| A | 1.00 |  | 1.00 |  |
| B | 8.74 (7.50-10.19) | <0.001 | 8.70 (7.46-10.15) | <0.001 |
| C | 7.47 (6.80-8.19) | <0.001 | 7.67 (6.97-8.43) | <0.001 |
| D | 0.16 (0.13-0.18) | <0.001 | 0.16 (0.13-0.18) | <0.001 |
| E | 5.56 (4.68-6.6) | <0.001 | 5.49 (4.62-6.53) | <0.001 |
| F | 10,260.34 (641.02-16,4229.60) | <0.001 | 10,470 (654.05-167,603.70) | <0.001 |
| G | 4.48 (4.07-4.93) | <0.001 | 4.70 (4.26-5.17) | <0.001 |
| H | 8.66 (6.67-11.24) | <0.001 | 9.57 (7.34-12.48) | <0.001 |
| I | 3.14 (2.85-3.45) | <0.001 | 3.15 (2.86-3.47) | <0.001 |
| J | 0.21 (0.18-0.24) | <0.001 | 0.20 (0.17-0.23) | <0.001 |
| K | 1.16 (1.07-1.26) | <0.001 | 1.16 (1.06-1.26) | 0.001 |
| L | 0.94 (0.84-1.05) | 0.280 | 0.95 (0.85-1.07) | 0.403 |

Analysis done using complete cases.

# **Multiple imputations**

Multiple imputation was conducted using a fully conditional specification(2) applied to all variables in the analysis model (VACS Index 2.0 components at follow-up start, and cohort), as well as the auxiliary variables (VACS Index 2.0 components at ART initiation). Using multiple imputation by chained equations (MICE), 100 imputations were done(3), with a burn-in of 200 iterations.

Linear regression was used to impute continuous variables and ordered logistic regression was used to impute ordered categorical variables. Multiple imputations were performed using Stata version 17.0(4).This work was carried out using the computational facilities of the Advanced Computing Research Centre, University of Bristol - <http://www.bristol.ac.uk/acrc/>.

# **Compatibility between the imputation model and the complete case model**

Before and after multiple imputation, the C-statistics were similar, with the confidence intervals overlapping for each cause-of-death (Supplementary Table 6). Deaths due to suicide/accident had the lowest discrimination in both models.

**Comparisons of regression models**

The HR and subdistribution hazard ratio (SHR) were similar for Cox and competing risk regression models (Supplementary Table 7). For comparison purposes, a user written command -somerd was used to estimate the C-statistics in both models.

**Sub-group analysis**

The C-statistics of the VACS Index 2.0 for all-cause mortality was similar between men and women (Supplementary Table 8), as well as between whites and non-whites (Supplementary Table 9).

**Supplementary Table 6. Comparison of complete case analysis and multiple imputation of missing data** **per 10-point increment of VACS Index 2.0**

|  | **Complete cases (N=9927)** | | | | **Imputed data (N=59741)** | | | |  |
| --- | --- | --- | --- | --- | --- | --- | --- | --- | --- |
| **Cause of death** | **Number of deaths** | **Mean VACS Index 2.0**  **(95% CI)** | **Hazard ratio**  **(95% CI)** | **C-statistic** | **Number of deaths** | **Mean VACS Index 2.0 (95% CI)** | **Hazard ratio**  **(95% CI)** | **C-statistic** | |
| All-cause | 437 | 68 (66-70) | 1.79 (1.72-1.86) | 0.83 | 2,425 | 68 (67-68) | 1.84 (1.81-1.87) | 0.83 | |
| AIDS | 94 | 78 (74-83) | 2.14 (1.96-2.34) | 0.89 | 455 | 78 (76-80) | 2.14 (2.06-2.23) | 0.91 | |
| Liver (including HCC) | 29 | 75 (67-84) | 2.06 (1.75-2.42) | 0.88 | 148 | 76 (72-80) | 2.13 (1.99-2.29) | 0.91 | |
| Cardiovascular | 38 | 62 (56-69) | 1.61 (1.40-1.85) | 0.79 | 214 | 62 (59-65) | 1.66 (1.56-1.77) | 0.79 | |
| Respiratory | 20 | 79 (70-87) | 2.20 (1.81-2.69) | 0.93 | 99 | 72 (68-75) | 2.00 (1.83-2.18) | 0.89 | |
| Non-AIDS infection | 22 | 70 (61-79) | 1.87 (1.57-2.23) | 0.81 | 141 | 72 (68-76) | 1.98 (1.85-2.13) | 0.87 | |
| Non-AIDS defining cancers | 80 | 66 (61-70) | 1.70 (1.54-1.86) | 0.83 | 452 | 66 (64-68) | 1.81 (1.73-1.89) | 0.83 | |
| Other | 49 | 67 (60-73) | 1.76 (1.56-1.99) | 0.80 | 306 | 66 (63-69) | 1.76 (1.67-1.85) | 0.78 | |
| Substance abuse | 25 | 57 (50-64) | 1.55 (1.31-1.85) | 0.79 | 97 | 62 (58-66) | 1.70 (1.56-1.87) | 0.83 | |
| Suicide/accident | 27 | 45 (38-53) | 1.13 (0.92-1.38) | 0.55 | 141 | 50 (46-54) | 1.32 (1.20-1.44) | 0.65 | |
| Unclassifiable | 53 | 64 (59-70) | 1.68 (1.49-1.88) | 0.81 | 372 | 63 (61-65) | 1.70 (1.62-1.78) | 0.77 | |

HCC-hepatocellular carcinoma.

**Supplementary Table 7. Comparison of Cox regression and competing risk regression models per 10-point increment of VACS Index 2.0 using complete cases**

|  |  | **Cox regression** | | **Competing risks regression** | |
| --- | --- | --- | --- | --- | --- |
| **Cause of death** | **Dead** | **HR (95% CI)** | **C-statistics (95% CI)** | **SHR (95% CI)** | **C-statistics (95% CI)** |
| All-cause | 437 | 1.79 (1.72-1.86) | 0.83 (0.80-0.85) | 1.81 (1.74-1.89) | 0.84 (0.82-0.86) |
| AIDS | 94 | 2.14 (1.96-2.34) | 0.90 (0.86-0.93) | 2.04 (1.88-2.21) | 0.92 (0.89-0.95) |
| Liver (including HCC) | 29 | 2.06 (1.75-2.42) | 0.89 (0.83-0.96) | 1.94 (1.70-2.21) | 0.92 (0.87-0.97) |
| Cardiovascular | 38 | 1.61 (1.40-1.85) | 0.79 (0.73-0.86) | 1.53 (1.38-1.70) | 0.81 (0.74-0.88) |
| Respiratory | 20 | 2.20 (1.81-2.69) | 0.94 (0.91-0.97) | 2.01 (1.73-2.34) | 0.96 (0.95-0.98) |
| Non-AIDS infection | 22 | 1.87 (1.57-2.23) | 0.85 (0.77-0.92) | 1.77 (1.54-2.04) | 0.84 (0.75-0.94) |
| Non-AIDS defining cancers | 80 | 1.70 (1.54-1.86) | 0.84 (0.80-0.88) | 1.61 (1.48-1.75) | 0.83 (0.78-0.88) |
| Other | 49 | 1.76 (1.56-1.99) | 0.82 (0.75-0.89) | 1.68 (1.50-1.88) | 0.84 (0.78-0.91) |
| Substance abuse | 25 | 1.55 (1.31-1.85) | 0.74 (0.65-0.84) | 1.46 (1.30-1.63) | 0.89 (0.85-0.94) |
| Suicide/accident | 27 | 1.13 (0.92-1.38) | 0.51 (0.39-0.62) | 1.08 (0.91-1.28) | 0.73 (0.65-0.82) |
| Unclassifiable | 53 | 1.68 (1.49-1.88) | 0.81 (0.74-0.87) | 1.57 (1.44-1.71) | 0.89 (0.84-0.93) |

HCC-hepatocellular carcinoma. SHR-subdistribution hazard ratio

**Supplementary Table 8. Comparison of men and women** **per 10-point increment of VACS Index 2.0, using multiply imputed data**

|  | **Men (N=47842)** | | | | **Women (N=** **11899)** | | | |  |
| --- | --- | --- | --- | --- | --- | --- | --- | --- | --- |
| **Cause of death** | **Number of deaths** | **Mean VACS Index 2.0 (95% CI)** | **Hazard ratio**  **(95% CI)** | **C-statistic** | **Number of deaths** | **Mean VACS Index 2.0 (95% CI)** | **Hazard ratio**  **(95% CI)** | **C-statistic** | |
| All-cause | 1971 | 68 (67-69) | 1.85 (1.81-1.89) | 0.83 | 454 | 68 (66-70) | 1.82 (1.74-1.90) | 0.82 | |
| AIDS | 354 | 77 (75-79) | 2.11 (2.02-2.20) | 0.92 | 101 | 80 (76-84) | 2.30 (2.09-2.53) | 0.89 | |
| Liver (including HCC) | 122 | 76 (72-80) | 2.13 (1.97-2.29) | 0.91 | 26 | 75 (65-84) | 2.22 (1.84-2.69) | 0.91 | |
| Cardiovascular | 173 | 62 (59-65) | 1.69 (1.58-1.81) | 0.79 | 41 | 60 (54-66) | 1.56 (1.33-1.84) | 0.77 | |
| Respiratory | 88 | 73 (68-77) | 2.05 (1.87-2.24) | 0.90 | 11 | 62 (52-72) | 1.55 (1.16-2.08) | 0.74 | |
| Non-AIDS infection | 109 | 74 (69-78) | 2.06 (1.91-2.22) | 0.88 | 32 | 65 (58-72) | 1.68 (1.43-1.99) | 0.81 | |
| Non-AIDS defining cancers | 377 | 66 (64-68) | 1.82 (1.74-1.91) | 0.83 | 75 | 66 (61-70) | (1.78 (1.59-2.00) | 0.81 | |
| Other | 251 | 66 (63-69) | 1.77 (1.67-1.87) | 0.79 | 55 | 66 (59-72) | 1.70 (1.50-1.93) | 0.76 | |
| Substance abuse | 74 | 63 (58-68) | 1.74 (1.57-1.92) | 0.83 | 23 | 59 (50-68) | 1.58 (1.28-1.94) | 0.81 | |
| Suicide/accident | 123 | 48 (45-52) | 1.28 (1.17-1.42) | 0.65 | 18 | 61 (49-73) | 1.64 (1.31-2.05) | 0.74 | |
| Unclassifiable | 300 | 64 (61-66) | 1.73 (1.64-1.82) | 0.77 | 72 | 62 (57-67) | 1.59 (1.42-1.79) | 0.77 | |

HCC-hepatocellular carcinoma.

**Supplementary Table 9. Comparison of ethnicity per 10-point increment of VACS Index 2.0, using multiply imputed data**

|  | **White (N=45666)** | | | | **Non-white (N=13252)** | | | | |  |
| --- | --- | --- | --- | --- | --- | --- | --- | --- | --- | --- |
| **Cause of death** | **Number of deaths** | **Mean VACS Index 2.0 (95% CI)** | **Hazard ratio**  **(95% CI)** | **C-statistic** | **Number of deaths** | **Mean VACS Index 2.0 (95% CI)** | **Hazard ratio**  **(95% CI)** | **C-statistic** | | |
| All-cause | 1954 | 67 (66-68) | 1.83 (1.79-1.87) | 0.83 | 423 | 71 (69-73) | 1.89 (1.81-1.98) | | 0.82 | |
| AIDS | 331 | 77 (74-79) | 2.11 (2.01-2.21) | 0.91 | 120 | 80 (77-84) | 2.23 (2.05-2.43) | | 0.91 | |
| Liver (including HCC) | 132 | 75 (71-79) | 2.11 (1.96-2.27) | 0.90 | 12 | 89 (77-102) | 2.53 (1.89-3.39) | | 0.96 | |
| Cardiovascular | 173 | 61 (58-64) | 1.65 (1.54-1.77) | 0.79 | 35 | 65 (57-72) | 1.68 (1.43-1.96) | | 0.76 | |
| Respiratory | 88 | 72 (68-76) | 2.02 (1.85-2.21) | 0.89 | 10 | 66 (55-76) | 1.62 (1.20-2.19) | | 0.84 | |
| Non-AIDS infection | 114 | 71 (67-76) | 1.97 (1.83-2.13) | 0.88 | 25 | 74 (65-83) | 2.07 (1.73-2.46) | | 0.79 | |
| Non-AIDS defining cancers | 370 | 66 (64-68) | 1.80 (1.72-1.89) | 0.83 | 71 | 70 (65-75) | 1.85 (1.65-2.06) | | 0.84 | |
| Other | 259 | 66 (63-68) | 1.75 (1.66-1.85) | 0.79 | 32 | 71 (62-80) | 1.86 (1.58-2.18) | | 0.78 | |
| Substance abuse | 88 | 62 (57-66) | 1.69 (1.54-1.86) | 0.82 | 8 | 65 (47-83) | 1.65 (1.23-2.22) | | 0.87 | |
| Suicide/accident | 120 | 50 (46-54) | 1.33 (1.20-1.46) | 0.65 | 20 | 51 (40-61) | 1.27 (0.99-1.62) | | 0.67 | |
| Unclassifiable | 279 | 63 (60-65) | 1.71 (1.61-1.80) | 0.78 | 90 | 65 (60-70) | 1.67 (1.51-1.85) | | 0.74 | |

HCC-hepatocellular carcinoma.

**References**

1. Firth D. Bias reduction of maximum likelihood estimates. Biometrika. 1993;80(1):27-38.

2. van Buuren S. Multiple imputation of discrete and continuous data by fully conditional specification. Stat Methods Med Res. 2007;16(3):219-42.

3. White IR, Royston P, Wood AM. Multiple imputation using chained equations: Issues and guidance for practice. Stat Med. 2011;30(4):377-99.

4. StataCorp. Stata Statistical Software: Release 17. College Station, TX: StataCorp LLC.2021.
